# Invariance properties for the error function used for multilinear regression

**DOI:** 10.1371/journal.pone.0208793

**Published:** 2018-12-26

**Authors:** Mark H. Holmes, Michael Caiola

**Affiliations:** 1 Department of Mathematical Sciences, Rensselaer Polytechnic Institute, Troy, New York, United States of America; 2 Yerkes National Primate Laboratory/Udall Center, Emory University, Atlanta, Georgia, United States of America; Universitat de Valencia, SPAIN

## Abstract

The connections between the error function used in multilinear regression and the expected, or assumed, properties of the data are investigated. It is shown that two of the most basic properties often required in data analysis, scale and rotational invariance, are incompatible. With this, it is established that multilinear regression using an error function derived from a geometric mean is both scale and reflectively invariant. The resulting error function is also shown to have the property that its minimizer, under certain conditions, is well approximated using the centroid of the error simplex. It is then applied to several multidimensional real world data sets, and compared to other regression methods.

## Introduction

The problem considered here concerns the modeling assumptions made in multilinear regression, and their role in determining the error function. To provide a simple example, a principal component analysis (PCA) is used to find low dimensional subspace approximations of a data set. It has the property that if the data set is rotated, and a PCA is used, the rotated versions of the same low dimensional subspace approximations are obtained. This means that a PCA is rotationally invariant. This is one of the reasons that it is often used in face recognition [1, 2], visual tracking [3], and other pattern recognition problems.

In contrast, linear least squares is not rotationally invariant. However, unlike a PCA, it is scale invariant. What this means is that if you scale the variables in the data set, the resulting minimizer is the scaled version of what is obtained for the unscaled case (this is explained more precisely in the next section). Scale invariance is important, for example, if you want your minimizer to be independent of what units are used (e.g., inches versus centimeters). The fact that a PCA is scale dependent, and that it is possible to be fairly sensitive to the scaling, is well-known [4, 5].

A third type of invariance, which will play a central role in this paper, concerns the order the variables are listed or labeled. Specifically, if the minimizer is unaffected by the reordering of the variables, the result is said to be reflectively invariant. A simple example of where this is important is edge detection, where the minimizer should be unaffected by which axis is labeled *x*, *y* or *z*. It is not hard to show that a PCA is reflectively invariant, but that linear least squares is not. It needs to be pointed out that there are different forms of reflective invariance depending on the hyperplane used for the reflection. As an example, in computer vision it is desirable to be able to recognize an object regardless of whether you are looking at it, or at its horizontal reflection as seen when looking in a mirror [6]. In this case the reflection is through the vertical plane. There is also some variation in how to refer to reflective invariance as used here. As a case in point, it has been referred to as neutral data fitting because, for this form of invariance, the variables must be treated symmetrically [7, 8].

**Fig 1:**
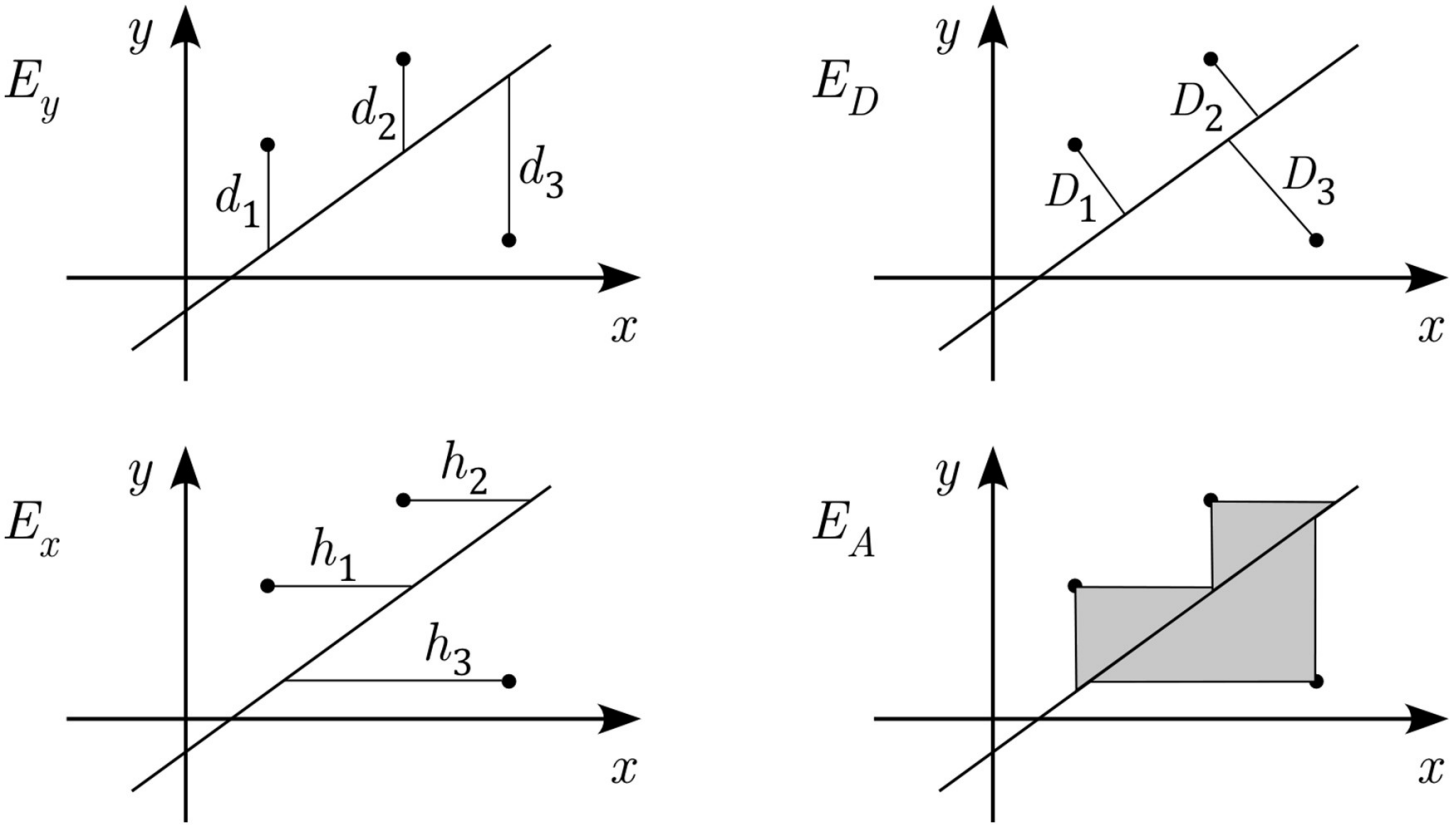
Error functions for bivariate regression. Shown are: $E_{y}=\sum d_{i}^{2}$, $E_{D}=\sum D_{i}^{2}$, $E_{x}=\sum h_{i}^{2}$, and $E_{A}=\frac{1}{2}\sum d_{i}h_{i}$.

Obtaining a regression result with particular invariance properties has been considered earlier, although often framed in somewhat different language. One of the earliest studies concerned reflective invariance for a bivariate problem using area as a measure of error [9]. This is the *E*_*A*_ example illustrated in Fig 1. This was the impetus for Samuelson [10] to propose certain invariant properties one might expect, or require, of the error function. Since then numerous attempts have been made to find error functions that have one or more of these properties. An example of a straightforward approach for two variable linear regression is to concentrate on the slope of the regression line. It has been proposed that, after determining the lines for vertical and horizontal least squares, *E*_*y*_ and *E*_*x*_ in Fig 1, to simply use the line whose slope is determined using an averaging method involving the two slopes. A review and comparison of these, and similar methods, can be found in [11, 12].

A more fundamental approach is to concentrate on the error function. One possibility is to use true distance, *E*_*D*_ in Fig 1, and this is easily generalized to multilinear regression (a PCA is an example). Another possibility is to use area, *E*_*A*_ in Fig 1, which is what Woolley used, and this gives rise to what is called least product regression, or least area regression. Work has been done on how to generalize Woolley’s idea, and use symmetry methods to obtain invariant error functions in two and three dimensions [7, 8]. However, this is not easily generalized to multilinear regression. An alternative, which is most relevant to the present study, is to use the geometric mean for each data point and then find the least squares value for this function [13]. This differs from the geometric mean considered here, which involves the geometric mean of the ordinary least squares error functions. The exception to this statement occurs when a hyperplane approximation is used, in which case the two formulations are equivalent. In the current study a hyperplane approximation is considered, but so are the other lower dimensional approximations that are possible (similar to what is done using a PCA).

In the next section it is shown that scale and rotational invariance are incompatible. This is done, for the case of two variables, by first characterizing mathematically what is needed to obtain particular invariant properties, and then to use similarity methods to demonstrate the incompatibility. In addition, in this two-dimensional setting, an error function that has several important invariance properties is formulated and discussed. In the subsequent two sections, the extension of this function to multilinear linear regression problems is considered, which includes showing that the minimizer is well-approximated using the centroid of an error simplex. With this, the error function is used to analyze real world data sets and compared to other regression methods.

## Two variables

To begin, we start with the case of two variables. Assume that the data are (*x*_1_, *y*_1_), (*x*_2_, *y*_2_), ⋯, (*x*_*n*_, *y*_*n*_), and they are centered. This means that ∑*x*_*i*_ = ∑*y*_*i*_ = 0. It is assumed in what follows that the data vectors **x** = (*x*_1_, *x*_2_, ⋯, *x*_*n*_)^*T*^ and **y** = (*y*_1_, *y*_2_, ⋯, *y*_*n*_)^*T*^ are not orthogonal.

For the model function *y* = *αx*, there are numerous ways to measure the error and four possibilities are shown in Fig 1. For a PCA the true distance is used, and this leads to the error function

$$\begin{array}{cc} E_{D}\left(\alpha \right) & =\sum_{i=1}^{n}D_{i}^{2}\\ & =\frac{1}{1+\alpha^{2}}\sum_{i=1}^{n}{\left(\alpha x_{i}-y_{i}\right)}^{2}\\ & =\frac{1}{1+\alpha^{2}}\left(\alpha^{2}x\cdot x-2\alpha x\cdot y+y\cdot y\right). \end{array}$$

Because of the denominator, for this expression to be defined, *α* must be dimensionless. What this means is that, when *x* and *y* have different dimensions, it is first necessary to nondimensionalize the variables before writing down the formula for the error function. So, suppose the data are scaled as *X*_*i*_ = *x*_*i*_/*S*_*x*_ and *Y*_*i*_ = *y*_*i*_/*S*_*y*_, where *S*_*x*_ and *S*_*y*_ are positive. The model function is now $Y=\bar{\alpha }X$, and the corresponding error function is

$$\begin{array}{c} \frac{1}{1+{\bar{\alpha }}^{2}}\sum_{i=1}^{n}{\left(\bar{\alpha }X_{i}-Y_{i}\right)}^{2}. \end{array}$$

Minimizing this, and then transforming back to dimensional variables, one finds that

$$\begin{array}{c} \alpha =\bar{\alpha }\;\frac{S_{y}}{S_{x}}\;, \end{array}$$ (1)

where

$$\begin{array}{c} \bar{\alpha }=\frac{1}{2}(-\lambda \pm \sqrt{\lambda^{2}+4}), \end{array}$$ (2)

for

$$\begin{array}{c} \lambda =\frac{X\cdot X-Y\cdot Y}{X\cdot Y}\;. \end{array}$$ (3)

In the above expression, the + is used if **X** ⋅ **Y** > 0 and the − is used if **X** ⋅ **Y** < 0. What is evident from this calculation is that *α* depends on *S*_*x*_ and *S*_*y*_. Typical choices for these scaling factors include *S*_*x*_ = ||**x**||_∞_, $S_{x}={\left||x|\right|}_{2}/\sqrt{n}$, and *S*_*x*_ = ||**x**||_1_/*n* (with similar expressions for *S*_*y*_). Depending on the scatter in the data, the values of these quantities can be significantly different and this can result in rather dramatic differences in the corresponding value of *α*.

### Scale invariance

There is a simple test for scale invariance that comes from the above analysis. To derive it, in the original *x*, *y*-coordinates and using the model equation *y* = *αx*, whatever regression procedure is used will result in the slope *α* depending on the data. This is written as *α* = *α*(**x**, **y**). Using the scaling *X* = *x*/*S*_*x*_ and *Y* = *y*/*S*_*y*_, the model equation becomes $Y=\bar{\alpha }X$, where $\bar{\alpha }=\alpha S_{x}/S_{y}$. Now, scale invariance requires that $\bar{\alpha }=\alpha \left(X,Y\right)$. Combining these two results, the requirement for scale invariance is that

$$\begin{array}{c} \alpha \left(x,y\right)=\frac{S_{x}}{S_{y}}\alpha \left(x/S_{x},y/S_{y}\right), \end{array}$$ (4)

for any positive values of *S*_*x*_ and *S*_*y*_. Using the infinitesimal generators *S*_*x*_ = 1 + *ε* and *S*_*y*_ = 1 + *δ*, then the above equation takes the form [14]

$$\begin{array}{cc} \alpha (x,y) & =\frac{1+\varepsilon }{1+\delta }\alpha \left(x/\left(1+\varepsilon \right),y/\left(1+\delta \right)\right)\\ & =\alpha \left(x,y\right)+\varepsilon [\alpha \left(x,y\right)-\nabla_{x}\alpha \left(x,y\right)]-\delta [\alpha \left(x,y\right)+\nabla_{y}\alpha \left(x,y\right)]+\cdots , \end{array}$$

where ∇_*x*_ is the gradient in the **x** variables (and similarly for ∇_*y*_). The *O*(*ε*) and *O*(*δ*) requirements are that

$$\begin{array}{c} x\cdot \nabla_{x}\alpha \left(x,y\right)=-\alpha \left(x,y\right),\;\;\text{and}\;\;y\cdot \nabla_{y}\alpha \left(x,y\right)=\alpha \left(x,y\right). \end{array}$$

These are easily solved using the *n*-dimensional version of spherical coordinates. Specifically, letting

$$\begin{array}{c} x=rX(\phi_{1},\phi_{2},\cdots ,\phi_{n-1}), \end{array}$$

where *r* = ||**x**||_2_ and the *ϕ*_*i*_’s are the angular coordinates, then **x** ⋅ ∇_*x*_ *α* = −*α* reduces to *r*∂_*r*_ *α* = −*α*. The general solution of this is *α* = *c*/*r*, where *c* can depend on the *ϕ*_*i*_’s. Doing something similar for **y**, the conclusion is that, to be scale invariant, the minimizer must depend on the data as

$$\begin{array}{c} \alpha =\frac{{\left||y|\right|}_{2}}{{\left||x|\right|}_{2}}F, \end{array}$$ (5)

where *F* can depend on the values of the angular coordinates for **x** and **y**.

### Rotational invariance

To determine the requirement for rotational invariance, assuming the angle of rotation is *θ*, then

$$\begin{array}{c} (\begin{array}{c} x^{\prime }\\ y^{\prime } \end{array})=(\;\;\begin{array}{cc} \cos\theta \;\; & \;\;\sin\theta \\ -\sin\theta \;\; & \;\;\cos\theta \end{array}\;)\;\;(\begin{array}{c} x\\ y \end{array}). \end{array}$$

The model function now has the form *y*′ = *α*′ *x*′, and invariance requires that

$$\begin{array}{c} \alpha^{\prime }=\frac{\alpha \cos\theta -\sin\theta }{\alpha \sin\theta +\cos\theta }\;. \end{array}$$

Writing the minimizer of the error function as *α* = *f*(**x**, **y**), then *α*′ = *f*(**x**′, **y**′) results in the requirement that

$$\begin{array}{c} \frac{f(x,y)\cos\theta -\sin\theta }{f(x,y)\sin\theta +\cos\theta }=f\left(x\cos\theta +y\sin\theta ,-x\sin\theta +y\cos\theta \right). \end{array}$$

Using the infinitesimal generator *θ* = *ε*, then the *O*(*ε*) requirement is

$$\begin{array}{c} y\cdot \nabla_{x}f-x\cdot \nabla_{y}f+f^{2}+1=0, \end{array}$$ (6)

where *f* = *f*(**x**, **y**). As it should, the solution in Eqs (2) and (3) satisfies this nonlinear partial differential equation. What does not satisfy the equation is Eq (5). It is easiest to illustrate this assuming there are only two data points. In this case, the 2-dimensional spherical coordinates used in Eq (5) can be written as *x*_1_ = *r* cos *k*, *x*_2_ = *r* sin *k*, *y*_1_ = *R* cos *K*, and *y*_2_ = *R* sin *K*. Substituting Eq (5) into Eq (6), and reducing gives

$$\begin{array}{c} (\frac{R^{2}}{r^{2}}+1)\cos\left(k-K\right)F+\sin\left(k-K\right)(\frac{R^{2}}{r^{2}}\partial_{k}F+\partial_{K}F)=1+\frac{R^{2}}{r^{2}}F^{2}. \end{array}$$

Given that *F* is independent of *r* and *R*, the above equation leads to the following two equations

$$\begin{array}{c} \cos\left(k-K\right)F+\sin\left(k-K\right)\partial_{k}F=F^{2}, \end{array}$$

and

$$\begin{array}{c} \cos\left(k-K\right)F+\sin\left(k-K\right)\partial_{K}F=1. \end{array}$$

These equations are solvable by introducing the change of variables *s* = *k* − *K*, *t* = *k* + *K*, and from this one finds that it is not possible to find a function *F* that satisfies both equations. In other words, there is no function which satisfies both Eqs (5) and (6).

### Scale and reflectively invariant error function

The conclusion from the above analysis is that it is not possible for the minimizer to be both scale and rotationally invariant. It is known that there are rotation and reflectively invariant error functions, and an example is one that uses true distance (*E*_*D*_ in Fig 1). So, the question considered here is whether there are error functions which satisfy all of the stated conditions except for rotational invariance.

It is relatively easy to find scale invariant error functions. For example, using the usual (vertical) least squares error

$$\begin{array}{c} E_{y}\left(\alpha \right)=\sum_{i=1}^{n}{\left(\alpha x_{i}-y_{i}\right)}^{2}=\alpha^{2}x\cdot x-2\alpha x\cdot y+y\cdot y, \end{array}$$ (7)

the minimum occurs when *α* = **x** ⋅ **y**/**x** ⋅ **x**. Similarly, using the (horizontal) least squares error

$$\begin{array}{c} E_{x}\left(\alpha \right)=\sum_{i=1}^{n}{\left(x_{i}-y_{i}/\alpha \right)}^{2}=x\cdot x-2x\cdot y/\alpha +y\cdot y/\alpha^{2}, \end{array}$$ (8)

the minimum occurs when *α* = **y** ⋅ **y**/**x** ⋅ **y**. Both of these are scale invariant. What *E*_*x*_ and *E*_*y*_ are not, however, are reflectively invariant. To be reflectively invariant it is required that irrespective of which variables are considered independent or dependent, that an equivalent result is obtained. This means that the minimizer of *E*_*x*_ is the same as the one obtained for *E*_*y*_. Mathematically, the requirement is that

$$\begin{array}{c} \alpha \left(y,x\right)=\frac{1}{\alpha (x,y)}\;. \end{array}$$ (9)

The error function to be considered here is based on the geometric mean of the ordinary least squares error functions. In the case of two variables, the error function is

$$\begin{array}{c} E\left(\alpha \right)=\sqrt{E_{x}\left(\alpha \right)E_{y}\left(\alpha \right)}\;, \end{array}$$ (10)

where *E*_*x*_ and *E*_*y*_ are given in Eqs (7) and (8), respectively. Minimizing this one finds that

$$\begin{array}{c} \alpha \left(x,y\right)=\pm \;\sqrt{\frac{y\cdot y}{x\cdot x}}\;, \end{array}$$ (11)

where the + is used if **x** ⋅ **y** > 0 and the − is used if **x** ⋅ **y** < 0. This satisfies the change of variables condition Eq (4), and this guarantees *α* is scale invariant. It is also reflectively invariant because it satisfies Eq (9). Another way to conclude that it is reflectively invariant is to note that the error function Eq (10) is a symmetric function of *E*_*x*_ and *E*_*y*_.

The minimizer in Eq (11) is well-known and can be obtained in a number of ways. This includes using geometric mean regression [9], using the geometric means of the minimizers for Eqs (7) and (8), and by using simple symmetry arguments [10]. What is not known is a way to generalize it to multilinear regression to obtain scale and reflectively invariant low dimensional approximations of data. What is presented below is one way this might be possible.

For ordinary least squares, one of the standard measures on how well the linear model fits the data is the coefficient of determination *R*^2^. For the multidimensional generalizations of Eq (10) considered later, a natural measure of fit involves the centroid of the error simplex. To explain what this is for this two dimensional problem, and connect it with *R*, let *α*_*x*_ and *α*_*y*_ be the minimizers of *E*_*x*_ and *E*_*y*_, respectively. The centroid in this case is *α*_*c*_ = (*α*_*x*_ + *α*_*y*_)/2. The error measure to be introduced concerns how *α*, the minimizer of *E*, differs from the centroid, relative to the width of the simplex. The resulting formula is

$$\begin{array}{c} C_{F}=|\frac{\alpha -\alpha_{c}}{\alpha_{y}-\alpha_{x}}|. \end{array}$$

Now, using the law of cosines **x** ⋅ **y** = ||**x**||_2_ ⋅ ||**y**||_2_cos *θ*, where *θ* is the angle between **x** and **y**, then *α*_*x*_ = *α*/cos *θ* and *α*_*y*_ = *α* cos *θ*. Moreover, *R*^2^ = cos^2^ *θ*, which gives a (signed) value of *R* = cos *θ*. Combining these formulas, the result is

$$\begin{array}{c} C_{F}=\frac{1}{2}\cdot \frac{1-|R|}{1+|R|}\;. \end{array}$$

Consequently, the better the fit (the closer *R*^2^ is to one), the closer the minimizer of *E* is to the centroid of the error simplex formed from the minimizers of *E*_*x*_ and *E*_*y*_.

## Multilinear regression: Single component approximation

It is now assumed that there are *m* variables, so **p** = (*p*_1_, *p*_2_, ⋯, *p*_*m*_)^*T*^. The centered data vectors for each variable are **p**_1_ = (*p*_11_, *p*_12_, ⋯, *p*_1*n*_)^*T*^, **p**_2_ = (*p*_21_, *p*_22_, ⋯, *p*_2*n*_)^*T*^, ⋯, **p**_*m*_ = (*p*_*m*1_, *p*_*m*2_, ⋯, *p*_*mn*_)^*T*^. It is assumed that no two of these vectors are orthogonal.

One of the central components of a PCA is the ability to find low dimensional approximations of the form **p** = *α*_1_**v**_1_ + ⋯+ *α*_*k*_**v**_*k*_, where 1 ≤ *k* < *m*. The question is whether something similar can be done for a scale and reflectively invariant approximation. One possibility, which is pursued here, is to use the geometric mean of the ordinary least squares functions.

We begin with a one dimensional subspace, which means that **p** = *α***v**. In what follows this is rewritten as *p*_2_ = *α*_2_*p*_1_, *p*_3_ = *α*_3_*p*_1_, ⋯, *p*_*m*_ = *α*_*m*_*p*_1_. The corresponding individual error functions are then

$$\begin{array}{cc} E_{1} & =\sum_{i=1}^{n}[{\left(p_{1i}-p_{2i}/\alpha_{2}\right)}^{2}+{\left(p_{1i}-p_{3i}/\alpha_{3}\right)}^{2}+\cdots +{\left(p_{1i}-p_{mi}/\alpha_{m}\right)}^{2}],\\ E_{2} & =\sum_{i=1}^{n}[{\left(p_{2i}-\alpha_{2}p_{1i}\right)}^{2}+{\left(p_{2i}-\alpha_{2}p_{3i}/\alpha_{3}\right)}^{2}+\cdots +{\left(p_{2i}-\alpha_{2}p_{mi}/\alpha_{m}\right)}^{2}],\\ \vdots \;\;\;\; & =\;\;\vdots \\ E_{m} & =\sum_{i=1}^{n}[{\left(p_{mi}-\alpha_{m}p_{1i}\right)}^{2}+{\left(p_{mi}-\alpha_{m}p_{2i}/\alpha_{2}\right)}^{2}+\cdots +{\left(p_{mi}-\alpha_{m}p_{m-1,i}/\alpha_{m-1}\right)}^{2}]. \end{array}$$

Letting *α*_1_ = 1, the general form of the above can be written as

$$\begin{array}{cc} E_{j} & =\sum_{i=1}^{n}[{\left(p_{ji}-\alpha_{j}p_{1i}/\alpha_{1}\right)}^{2}+{\left(p_{ji}-\alpha_{j}p_{2i}/\alpha_{2}\right)}^{2}+\cdots +{\left(p_{ji}-\alpha_{j}p_{mi}/\alpha_{m}\right)}^{2}]\\ & =p_{j}\cdot p_{j}-2\alpha_{j}p_{j}\cdot p_{1}+\alpha_{j}^{2}p_{1}\cdot p_{1}/\alpha_{1}^{2}+\cdots \\ & \;\;+p_{j}\cdot p_{j}-2\alpha_{j}p_{j}\cdot p_{m}+\alpha_{j}^{2}p_{m}\cdot p_{m}/\alpha_{m}^{2}. \end{array}$$ (12)

After factoring, this can be written in the more compact form

$$\begin{array}{c} E_{j}=\sum_{k=1}^{m}(p_{j}-\frac{\alpha_{j}}{\alpha_{k}}p_{k})\cdot (p_{j}-\frac{\alpha_{j}}{\alpha_{k}}p_{k}). \end{array}$$ (13)

It is worth pointing out that if one of the *E*_*j*_’s is zero, then they are all zero.

The resulting error function, which comes from the geometric mean of the *E*_*j*_’s, is *E* = (*E*_1_ *E*_2_⋯*E*_*m*_)^1/*m*^. As stated earlier, this is reflectively invariant because of the symmetric dependence of *E* on the individual error functions.

To make the connection between the minimizer of *E* and the error centroid, the minimizer for each *E*_*j*_ is needed. Finding them is straightforward. Namely, setting the first partials of *E*_*j*_ to zero, one finds that

$$\begin{array}{c} \alpha_{j}=\frac{p_{j}\cdot p_{1}}{p_{1}\cdot p_{1}}\;, \end{array}$$ (14)

and, for *i* ≠ *j*,

$$\begin{array}{c} \alpha_{i}=\frac{p_{i}\cdot p_{i}}{p_{j}\cdot p_{i}}\;\alpha_{j}. \end{array}$$ (15)

Note that these use the stated assumption that the data vectors are not orthogonal. Also, the above expressions hold in the case of when *j* = 1 because *α*_1_ = 1.

Assume now that the data are close to linear, which means that **p**_*i*_ = *α*_*i*0_**p**_10_ + *ε***p**_*i*1_, for *i* = 1, 2, ⋯, *m*. Given a value for *j* in Eq (13), the corresponding asymptotic expansions of its minimizing coefficients, for small *ε*, have the form

$$\begin{array}{c} \alpha_{i}∼\alpha_{i0}+\varepsilon \alpha_{i1}+\varepsilon^{2}\alpha_{i2}+\cdots ,\;\;\text{for}\;i=1,2,\cdots ,m. \end{array}$$

Note that in the case *i* = 1, the coefficients are *α*_10_ = 1 and *α*_11_ = *α*_12_ = 0. The *α*_*i*0_’s are assumed to be known, and the other coefficients are determined by minimizing the error. In preparation for this, note that

$$\begin{array}{cc} p_{j}-\frac{\alpha_{j}}{\alpha_{k}}p_{k} & ∼\alpha_{i0}p_{10}+\varepsilon p_{i1}-\frac{\alpha_{i0}+\varepsilon \alpha_{i1}}{\alpha_{k0}+\varepsilon \alpha_{k1}}(\alpha_{k0}p_{10}+\varepsilon p_{k1})\\ & ∼\varepsilon \alpha_{j0}\left(q_{j}-q_{k}\right), \end{array}$$

where (*i* = *j*, *k*)

$$\begin{array}{c} q_{i}=\frac{1}{\alpha_{i0}}(p_{i1}-\alpha_{i1}p_{10}). \end{array}$$

From Eq (13) it follows that

$$\begin{array}{c} E_{j}∼\frac{1}{\alpha_{j0}^{2}}\varepsilon^{2}\sum_{k=1}^{m}(q_{j}-q_{k})\cdot (q_{j}-q_{k}). \end{array}$$

Minimizing this determines the *α*_*i*1_’s, and this yields

$$\begin{array}{c} \alpha_{i}∼\alpha_{i0}+\varepsilon \frac{p_{10}\cdot \left(p_{i1}-\alpha_{i0}p_{11}\right)}{p_{10}\cdot p_{10}}\;,\;\text{for}\;i=2,3,\cdots m. \end{array}$$ (16)

What is significant about this is that the first two terms in the expansions for the *α*_*i*_’s do not depend on *j*. In other words, to *O*(*ε*), the *E*_*j*_’s have the same minimizer. Because of this, it immediately follows that through terms of order *ε*, the minimizer of the error function *E* equals the centroid formed from the minimizers of the *E*_*j*_’s. Expressed mathematically, if ***α*** is the minimizer of *E*, and ***α***_*j*_ the minimizer of *E*_*j*_, then

$$\begin{array}{c} \alpha =\frac{1}{m}\sum_{j=1}^{m}\alpha_{j}+O\left(\varepsilon^{2}\right). \end{array}$$ (17)

It also follows from this analysis that, for small *ε*, the error function *E* is strictly convex in the neighborhood of the minimum.

### Example in $R^{3}$

**Fig 2:**
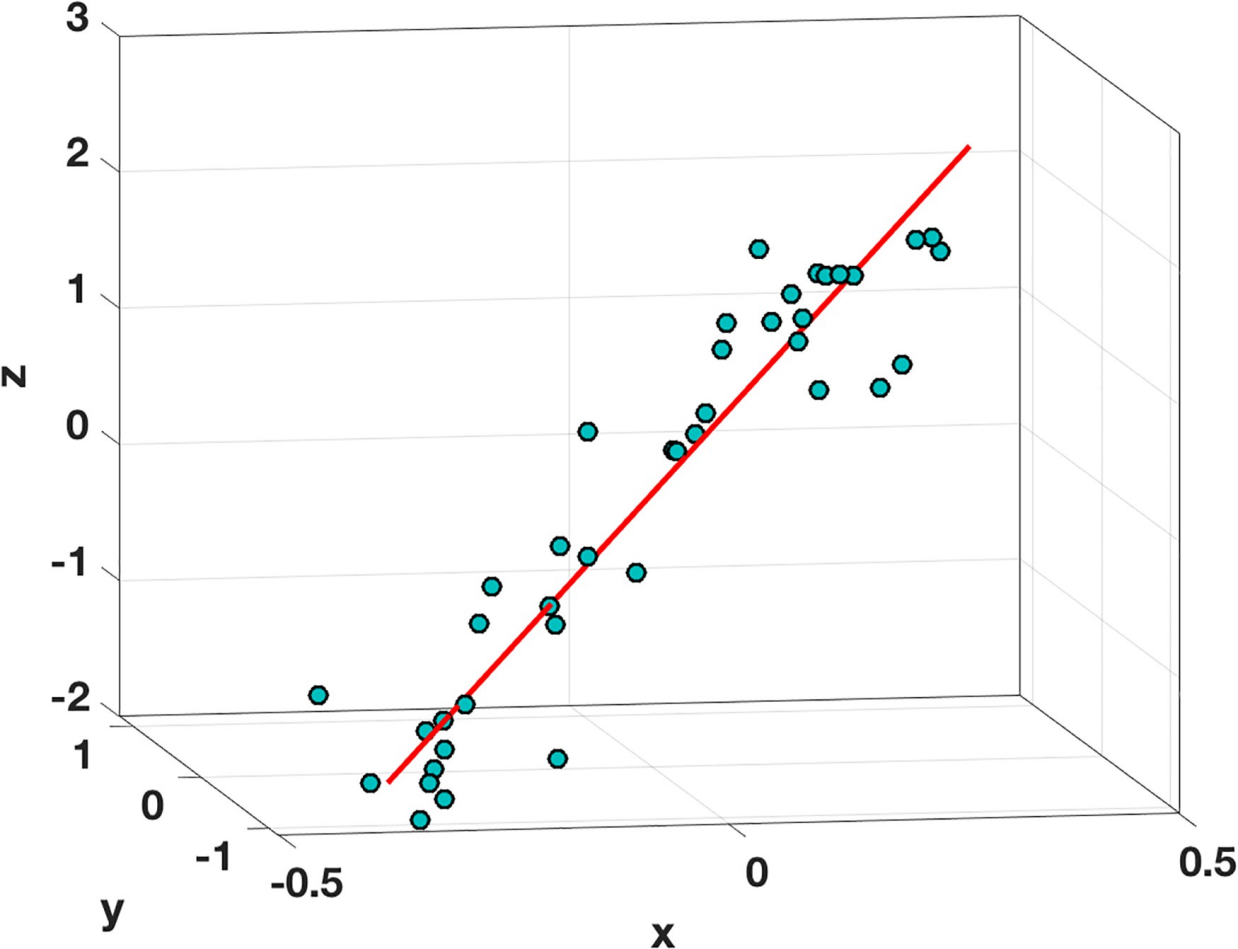
Data used for line fitting example in $R^{3}$. The red line is the linear fit determined by minimizing *E* = (*E*_*x*_*E*_*y*_*E*_*z*_)^1/3^.

**Fig 3:**
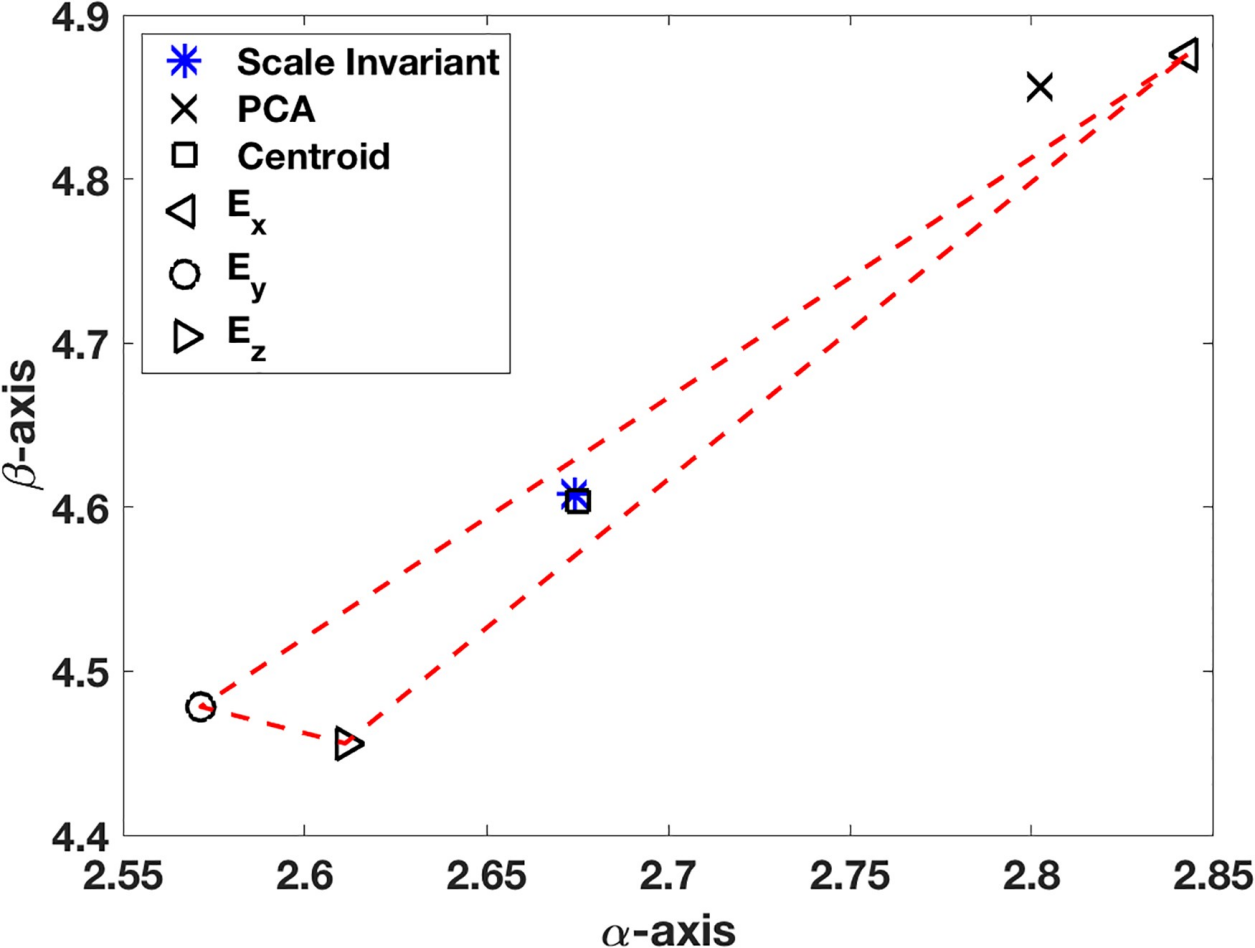
Location of minimizer for the error function *E* = (*E*_*x*_*E*_*y*_*E*_*z*_)^1/3^, as well as the location determined using an unscaled PCA. Vertices of the triangle (simplex) are the locations of the minimizers of *E*_*x*_, *E*_*y*_, and *E*_*z*_.

As an example, in $R^{3}$, for the line with *α*_2_ = 3 and *α*_3_ = 5, 40 randomized points within a distance of 0.2 of the line are used for the data (see Fig 2). For notational simplicity, let *p*_1_ = *x*, *p*_2_ = *y*, *p*_3_ = *z*, *α*_2_ = *α*, and *α*_3_ = *β*. The location of the minimizer of *E* was found using MATLAB’s *fminsearch* command, and the location is shown in Fig 3. Also shown are the locations of the minimizers for *E*_*x*_, *E*_*y*_, and *E*_*z*_, determined by Eqs (14) and (15), as well as the associated triangular region formed by these three points. For comparison, the location of the solution as determined using an unscaled PCA is shown. An important observation coming from this figure is that the minimizer for the scale (and reflectively) invariant error function *E* = (*E*_*x*_*E*_*y*_*E*_*z*_)^1/3^ is located near the centroid of the triangle. This is expected because of Eq (17).

It is worth having a way to characterize how close the minimizer and centroid are, to provide a measure for the goodness of fit. One possibility is to use the maximum distance relative to the width of the simplex, as given by the formula

$$\begin{array}{c} C_{F}=\max\{\frac{|\alpha -\alpha_{c}|}{w_{\alpha }}\;,\frac{|\beta -\beta_{c}|}{w_{\beta }}\}, \end{array}$$ (18)

where (*α*_*c*_, *β*_*c*_) is the centroid, *w*_*α*_ = max{*α*_*x*_, *α*_*y*_, *α*_*z*_} − min{*α*_*x*_, *α*_*y*_, *α*_*z*_}, *w*_*β*_ = max{*β*_*x*_, *β*_*y*_, *β*_*z*_} − min{*β*_*x*_, *β*_*y*_, *β*_*z*_}. In these expressions, (*α*_*x*_, *β*_*x*_), (*α*_*y*_, *β*_*y*_), and (*α*_*z*_, *β*_*z*_) are the minimizers of *E*_*x*_, *E*_*y*_, and *E*_*z*_, respectively, and they determine the error simplex in Fig 3. For the solution shown in Fig 3, *C*_*F*_ ≈ 0.01.

**Fig 4:**
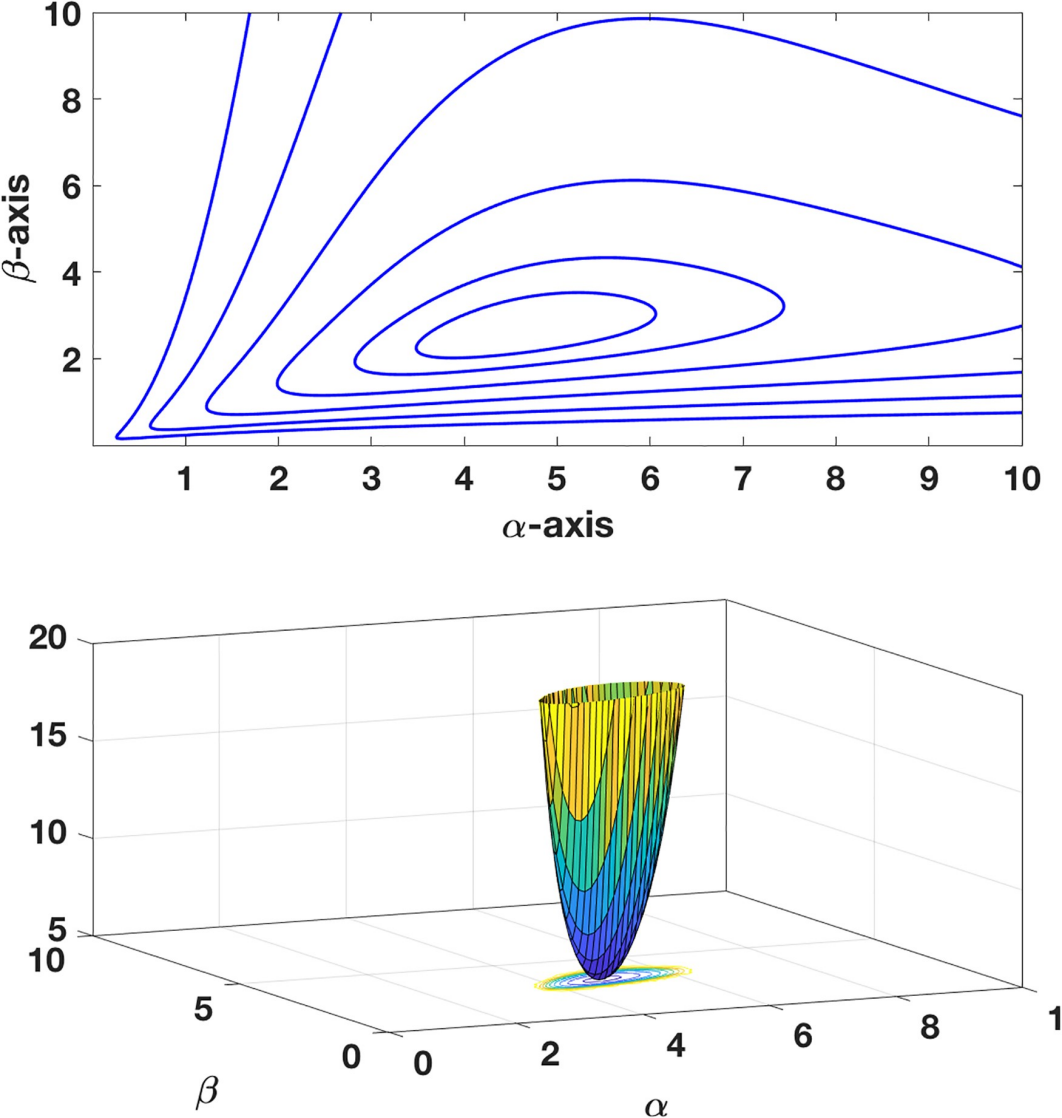
Contour and surface plots for error function *E* = (*E*_*x*_*E*_*y*_*E*_*z*_)^1/3^, using the the data in Fig 2.

Finally, in finding the minimizer the question of whether or not the error function is convex arises. To verify this, its contour and surface plot are shown in Fig 4. Note that, because of the assumed form of the model function in this example, the error functions *E*_*x*_, *E*_*y*_, and *E*_*z*_, and *E* are undefined when either *α* or *β* are zero. The statement that *E* is convex refers to its dependence on *α* and *β* in the quadrant in which the minimizers are located.

## Multilinear regression: (*m* − 1)-component approximation

The assumptions on the data are the same as for the one-dimensional approximation considered above. As for the model function, it is a hyperplane that is written as *p*_*m*_ = *α*_1_*p*_1_ + *α*_2_ *p*_2_+ ⋯+ *α*_*m*−1_ *p*_*m*−1_. The associated individual error functions are

$$\begin{array}{c} E_{j}=\frac{1}{\alpha_{j}^{2}}\sum_{i=1}^{n}{\left(p_{mi}-\alpha_{1}p_{1i}-\alpha_{2}p_{2i}-\cdots -\alpha_{m-1}p_{m-1,i}\right)}^{2},\;\;\text{for}\;j=1,2,\cdots ,m, \end{array}$$ (19)

where *α*_*m*_ = 1. With this, the composite error function is

$$\begin{array}{cc} E(\alpha_{1}, & \alpha_{2},\cdots ,\alpha_{m-1})={\left(E_{1}E_{2}\cdots E_{m}\right)}^{1/m}\\ & =\frac{1}{{\left(\alpha_{1}\alpha_{2}\cdots \alpha_{m-1}\right)}^{2/m}}\sum_{i=1}^{n}{\left(p_{mi}-\alpha_{1}p_{1i}-\alpha_{2}p_{2i}-\cdots -\alpha_{m-1}p_{m-1,i}\right)}^{2}. \end{array}$$ (20)

Letting ***α*** = (*α*_1_, *α*_2_, ⋯, *α*_*m*−1_, −1)^*T*^ and **P** = (**p**_1_ **p**_2_ ⋯ **p**_*m*_), then this error function can be written as

$$\begin{array}{c} E=\frac{1}{{\left(\alpha_{1}\alpha_{2}\cdots \alpha_{m-1}\right)}^{2/m}}{\left||P\alpha |\right|}_{2}^{2}. \end{array}$$ (21)

To obtain the connection between the minimizer of *E* and the centroid of the error simplex we need the minimizers for the *E*_*j*_’s. To determine them, note that for *k* = 1, 2, ⋯, *m* − 1,

$$\begin{array}{c} \partial_{\alpha_{k}}P\alpha =p_{k}. \end{array}$$

Also, from Eq (19), $E_{j}=P\alpha \cdot P\alpha /\alpha_{j}^{2}$. Consequently,

$$\begin{array}{c} \partial_{\alpha_{k}}E_{j}=\frac{2}{\alpha_{j}^{2}}P\alpha \cdot (p_{k}-\frac{\delta_{kj}}{\alpha_{j}}P\alpha ), \end{array}$$

where *δ*_*kj*_ is the Kronecker delta. To determine the equation to solve to find the minimizer, consider the case for *E*_1_. Setting ∇_*α*_*E*_1_ = **0**, yields the (*m* − 1) × (*m* − 1) system of equations

$$\begin{array}{c} A\alpha =(\begin{array}{c} P\alpha \cdot P\alpha /\alpha_{1}\\ 0\\ \vdots \\ 0 \end{array}), \end{array}$$ (22)

where **A** consists of the first *m* − 1 rows of **P**^*T*^**P**. Since **P*α*** ⋅ **P*α*** = ***α*** ⋅ (**P**^*T*^**P**)***α***, then, letting **r**^*T*^ be the *m*th row of **P**^*T*^**P**,

$$\begin{array}{c} P\alpha \cdot P\alpha =\alpha \cdot (\begin{array}{c} A\\ r^{T} \end{array})\alpha =P\alpha \cdot P\alpha -r\cdot \alpha . \end{array}$$

This means that Eq (22) can be replaced with the equation **R*α*** = **0**, where **R** is the matrix obtained by removing the first row from **P**^*T*^**P**. In a similar manner, the minimizer for *E*_*j*_ is found by solving the equation obtained by removing the *j*th row from **P**^*T*^**P**. To be more explicit about what equation needs to be solved, for *E*_1_, it is

$$\begin{array}{c} (\begin{array}{cccc} p_{1}\cdot p_{2}\; & \;p_{2}\cdot p_{2}\; & \;\cdots \; & \;p_{m-1}\cdot p_{2}\\ \vdots \; & \;\vdots \; & \;\cdots \; & \;\vdots \\ p_{1}\cdot p_{m-1}\; & \;p_{2}\cdot p_{m-1}\; & \;\cdots \; & \;p_{m-1}\cdot p_{m-1}\\ p_{1}\cdot p_{m}\; & \;p_{2}\cdot p_{m}\; & \;\cdots \; & \;p_{m-1}\cdot p_{m} \end{array})\;\;(\begin{array}{c} \alpha_{1}\\ \alpha_{2}\\ \vdots \\ \alpha_{m-1} \end{array})=(\begin{array}{c} p_{2}\cdot p_{m}\\ \vdots \\ p_{m-1}\cdot p_{m}\\ p_{m}\cdot p_{m} \end{array}). \end{array}$$

In general, the coefficient matrix for *E*_*j*_ is the (*m* − 1) × (*m* − 1) matrix that is obtained by removing the *j*th row and *m*th column from **P**^*T*^**P**, and the right hand side is the (*m* − 1)-vector that is obtained by removing the *j*th entry in the *m*th column of **P**^*T*^**P**.

To establish the connection between the centroid and the minimizer of *E*, assume that the data are close to planar. Specifically, letting **P** = **P**_0_ + *ε* **P**_1_, then for small *ε*, ***α*** ∼ ***α***_0_ + *ε**α***_1_ + *ε*^2^ ***α***_2_+ ⋯, where **P**_0_***α***_0_ = **0**. Now, setting ∇_*α*_ *E* = **0**, the problem to solve is

$$\begin{array}{c} A\alpha =\frac{1}{m}(\begin{array}{c} 1/\alpha_{1}\\ 1/\alpha_{2}\\ \vdots \\ 1/\alpha_{m-1} \end{array})P\alpha \cdot P\alpha . \end{array}$$ (23)

Also, **A** = **A**_0_ + *ε***A**_1_ + *ε*^2^**A**_2_ + ⋯, where **A**_0_, **A**_1_, **A**_2_ are the first (*m* − 1)-rows of $P_{0}^{T}P_{0}$, $P_{0}^{T}P_{1}+P_{1}^{T}P_{0}$, and $P_{1}^{T}P_{1}$, respectively. With this, the *O*(*ε*) problem that comes from Eq (23) is

$$\begin{array}{c} A_{0}\alpha_{1}+A_{1}\alpha_{0}=0, \end{array}$$ (24)

and the *O*(*ε*^2^) problem is

$$\begin{array}{c} A_{0}\alpha_{2}+A_{1}\alpha_{1}+A_{2}\alpha_{0}=\frac{1}{m}(\begin{array}{c} 1/\alpha_{10}\\ 1/\alpha_{20}\\ \vdots \\ 1/\alpha_{m-1,0} \end{array})(P_{0}\alpha_{1}+P_{1}\alpha_{0})\cdot (P_{0}\alpha_{1}+P_{1}\alpha_{0}). \end{array}$$ (25)

In comparison, using the same form for the expansions for *E*_1_, then one finds from Eq (22) that the *O*(*ε*) equation is the same as the one given in Eq (24). This is also true for the other *E*_*j*_’s. Therefore, the ***α***_1_ term for the centroid and for minimizer of *E* are equal (as is the ***α***_0_ term). As for the *O*(*ε*^2^) term, for *E*_1_, one finds from Eq (22) that the problem to solve is

$$\begin{array}{c} A_{0}\alpha_{2}+A_{1}\alpha_{1}+A_{2}\alpha_{0}=(\begin{array}{c} 1/\alpha_{10}\\ 0\\ \vdots \\ 0 \end{array})(P_{0}\alpha_{1}+P_{1}\alpha_{0})\cdot (P_{0}\alpha_{1}+P_{1}\alpha_{0}). \end{array}$$ (26)

The equations for the other *E*_*j*_’s are the same except for the appropriate modification of the first vector on the right hand side of the equation. Given that ***α***_0_ and ***α***_1_ are the same for *E* and the *E*_*j*_’s, it follows that the *O*(*ε*^2^) term in the centroid and the minimizer of *E* are equal. Therefore, the conclusion is that the minimizer of *E* and the centroid are equal through terms of order *ε*^2^. Expressed mathematically, if ***α*** is the minimizer of *E*, and ***α***_*j*_ the minimizer of *E*_*j*_, then

$$\begin{array}{c} \alpha =\frac{1}{m}\sum_{j=1}^{m}\alpha_{j}+O\left(\varepsilon^{3}\right). \end{array}$$ (27)

### Example in $R^{3}$

As before, for notational simplicity, let *p*_1_ = *x*, *p*_2_ = *y*, *p*_3_ = *z*, *α*_2_ = *α*, and *α*_3_ = *β*. The model function can then be written as *z* = *αx* + *βy*. In this case, from Eq (19), the individual error functions are:

$$\begin{array}{cc} E_{x} & =\sum_{i=1}^{n}{\left(x_{i}-z_{i}/\alpha +\beta y_{i}/\alpha \right)}^{2}\\ & =x\cdot x-2x\cdot z/\alpha +2\beta x\cdot y/\alpha +z\cdot z/\alpha^{2}-2\beta y\cdot z/\alpha^{2}+\beta^{2}y\cdot y/\alpha^{2}, \end{array}$$ (28)

$$\begin{array}{cc} E_{y} & =\sum_{i=1}^{n}{\left(y_{i}-z_{i}/\beta +\alpha x_{i}/\beta \right)}^{2}\\ & =y\cdot y-2y\cdot z/\beta +2\alpha x\cdot y/\beta +z\cdot z/\beta^{2}-2\alpha x\cdot z/\beta^{2}+\alpha^{2}x\cdot x/\beta^{2}, \end{array}$$ (29)

$$\begin{array}{cc} E_{z} & =\sum_{i=1}^{n}{\left(z_{i}-\alpha x_{i}-\beta y_{i}\right)}^{2}\\ & =z\cdot z-2\alpha x\cdot z-2\beta y\cdot z+\alpha^{2}x\cdot x+2\alpha \beta x\cdot y+\beta^{2}y\cdot y. \end{array}$$ (30)

The minimizer ***α***_*x*_ of *E*_*x*_ is

$$\begin{array}{c} \alpha_{x}=\frac{1}{\left(x\cdot y)(y\cdot z)-(x\cdot z)(y\cdot y\right)}(\;\;\begin{array}{cc} y\cdot z\;\; & \;-y\cdot y\\ -x\cdot z\;\; & \;x\cdot y \end{array}\;\;)(\begin{array}{c} y\cdot z\\ z\cdot z \end{array}), \end{array}$$ (31)

the minimizer ***α***_*y*_ of *E*_*y*_ is

$$\begin{array}{c} \alpha_{y}=\frac{1}{\left(x\cdot x)(y\cdot z)-(x\cdot z)(x\cdot y\right)}(\;\;\begin{array}{cc} y\cdot z\;\; & \;-x\cdot y\\ -x\cdot z\;\; & \;x\cdot x \end{array}\;\;)(\begin{array}{c} x\cdot z\\ z\cdot z \end{array}), \end{array}$$ (32)

and the minimizer ***α***_*z*_ of *E*_*z*_ is

$$\begin{array}{c} \alpha_{z}=\frac{1}{\left(x\cdot x\right)\left(y\cdot y\right)-{\left(x\cdot y\right)}^{2}}(\;\;\begin{array}{cc} y\cdot y\;\; & \;-x\cdot y\\ -x\cdot y\;\; & \;x\cdot x \end{array}\;\;)(\begin{array}{c} x\cdot z\\ y\cdot z \end{array}). \end{array}$$ (33)

The resulting error function based on the geometric mean is

$$\begin{array}{cc} E(\alpha ,\beta ) & ={\left(E_{x}E_{y}E_{z}\right)}^{1/3}\\ & =\frac{1}{\alpha^{2/3}\beta^{2/3}}(z\cdot z-2\alpha x\cdot z-2\beta y\cdot z+\alpha^{2}x\cdot x+2\alpha \beta x\cdot y+\beta^{2}y\cdot y). \end{array}$$ (34)

Taking the first partials of this, and setting them to zero, one obtains the (nonlinear) system

$$\begin{array}{cc} \alpha^{2}x\cdot x+\alpha \beta x\cdot y+\beta y\cdot z & =z\cdot z \end{array}$$ (35)

$$\begin{array}{cc} \beta^{2}y\cdot y+\alpha \beta x\cdot y+\alpha x\cdot z & =z\cdot z. \end{array}$$ (36)

These equations can be written in somewhat simpler terms by letting

$$\begin{array}{c} q=\alpha \sqrt{\frac{x\cdot x}{z\cdot z}}\;\text{and}\;p=\beta \sqrt{\frac{y\cdot y}{z\cdot z}}\;. \end{array}$$

Using the law of cosines, then Eqs (35) and (36) become

$$\begin{array}{cc} q^{2}+pq\cos\theta_{xy}+p\cos\theta_{yz} & =1 \end{array}$$ (37)

$$\begin{array}{cc} p^{2}+pq\cos\theta_{xy}+q\cos\theta_{xz} & =1, \end{array}$$ (38)

where *θ*_*xy*_ is the angle between **x** and **y** (with similar definitions for the other angles). An analytical formula for the solution of these equations is not apparent, but it is a simple matter to compute the solution numerically.

**Fig 5:**
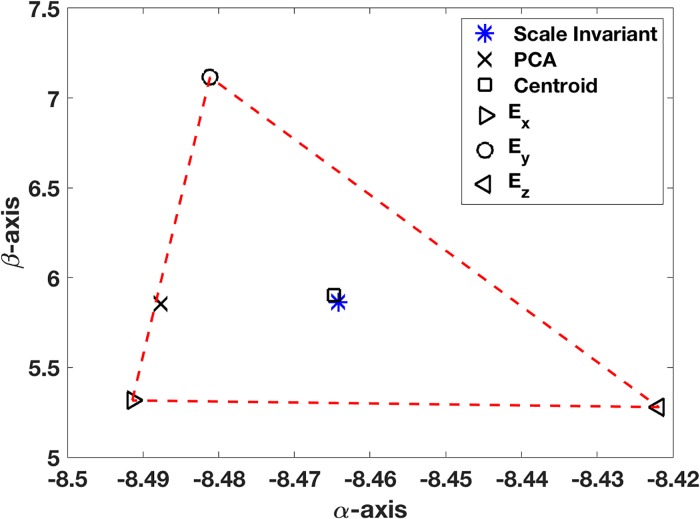
Typical locations for the various minimizers of the plane example. Vertices of the simplex are minimizers using individual coordinate projections *E*_*x*_, *E*_*y*_, and *E*_*z*_.

**Fig 6:**
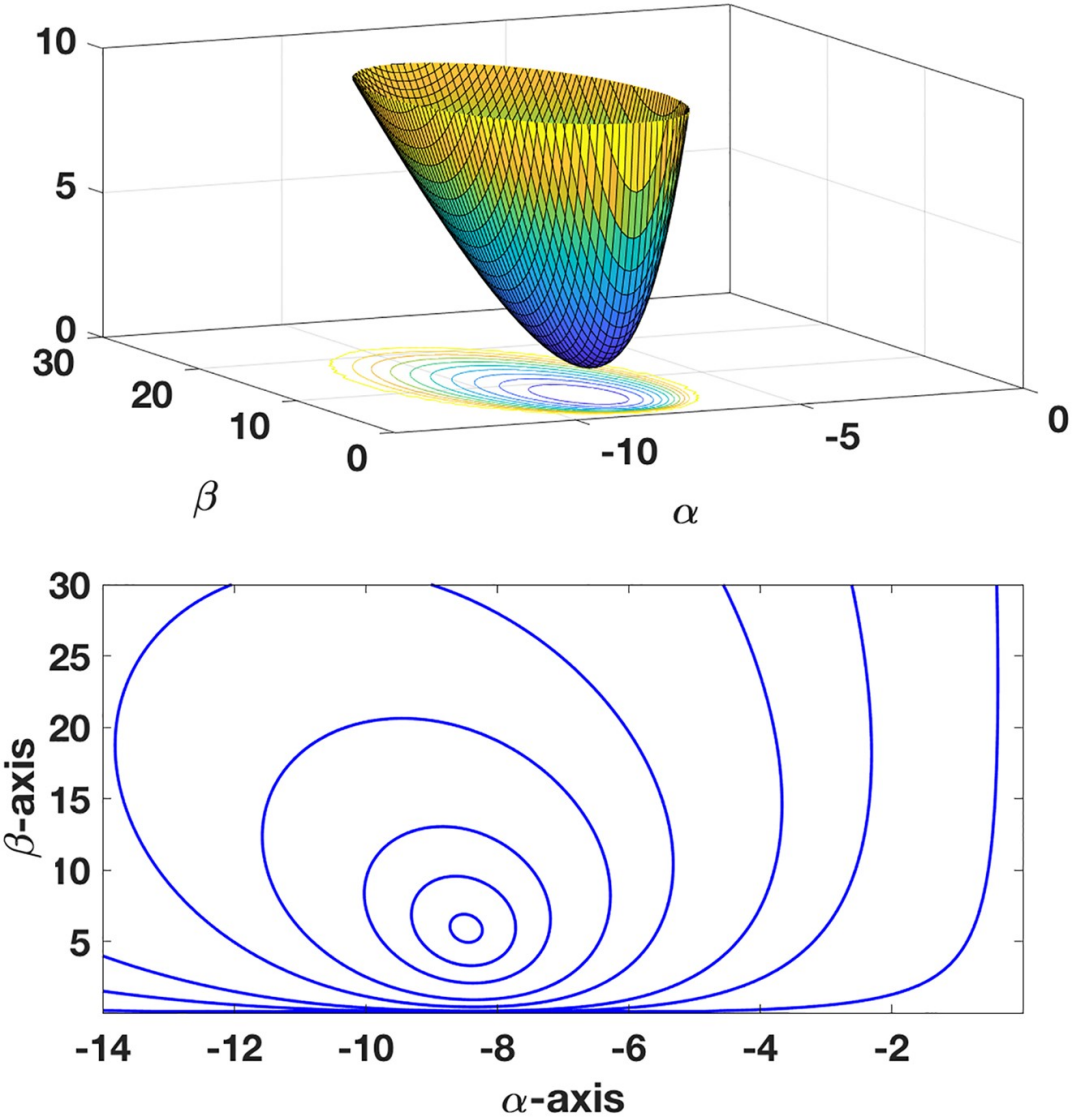
Contour and surface plots for error function *E* = (*E*_*x*_*E*_*y*_*E*_*z*_)^1/3^ for the planar fit data.

As an example, for the plane with *α* = −10 and *β* = 6, 40 randomized points within a distance of 0.15 of the plane were used for the data. The location of the minimizer of *E* was found by solving Eqs (35) and (36) using MATLAB’s *fminsearch* command, and the location is shown in Fig 5. Also shown are the locations of the minimizers for *E*_*x*_, *E*_*y*_, and *E*_*z*_, and the associated triangular region formed by these three points. For comparison, the location of the solution as determined using an unscaled PCA is shown. As expected from Eq (27), the of *E* minimizer is located near the centroid of the triangle. To quantify the difference, using the formula in Eq (18), *C*_*F*_ ≈ 0.02. Finally, to demonstrate the convexity of the error function in the octant containing the minimizer, the contour and surface are shown in Fig 6.

## Application to real world data sets

What follows are applications of the hyperplane function to various real world data sets. In the process, comparisons are made with a PCA. Also, three of the data sets have been used in other studies to compare multiple linear regression methods, and this is discussed in the respective application.

### Crime data

As an illustration, and a comparison with a PCA, consider the data for the seven major crime rates and population for the larger cities in the U.S in 2009 [15]. Of the 105 cities reported, 79 were randomly chosen for the training set, and the testing dataset consists of those left out. With this, *n* = 79 and *m* = 8. The minimizer of Eq (20) was found using a modified Polak-Ribière descent procedure, with Armijo’s method used to solve the line search problem. The modification is that if the search direction determined using Polak-Ribière is not a direction of descent, then the steepest descent direction is used instead. A description of the Polak-Ribière and Armijo’s methods can be found in [5, 16]. The starting point for the descent procedure was a convex combination of the minimizers for the *E*_*j*_’s, which was computed using the formulas given earlier. Also, the normalization for the PCA uses $\left|\right|p_{j}{\left|\right|}_{2}/\sqrt{n}$, for each column *j* of the centered training data matrix.

**Fig 7:**
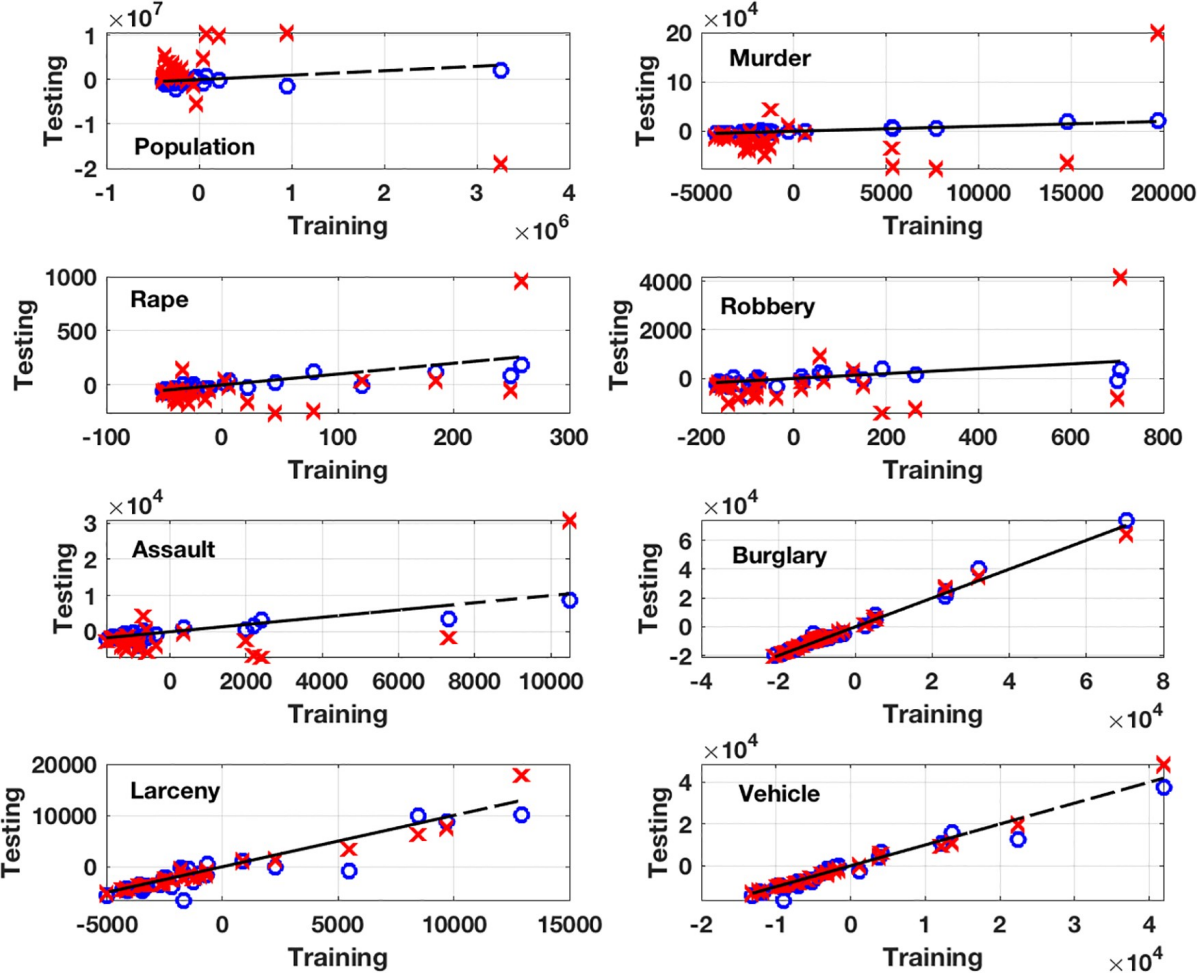
Comparison between fits using a hyperplane approximation to the crime and population data. Shown are the values obtained using the error function in Eq (20), indicated with the o’s, and using a PCA (x). The dashed line in each graph corresponds when the training and testing data are equal.

The resulting testing versus training comparison for each variable is shown in Fig 7. For Eq (20), the *α*_*j*_’s are determined by minimizing *E* and then using those same values for each graph. For example, for the graph associated with *p*_1_, the model function is rewritten as

$$\begin{array}{c} p_{1}=-\frac{\alpha_{2}}{\alpha_{1}}p_{2}-\frac{\alpha_{3}}{\alpha_{1}}p_{3}-\cdots -\frac{\alpha_{m-1}}{\alpha_{1}}p_{m-1}+\frac{1}{\alpha_{1}}p_{m}, \end{array}$$ (39)

and the resulting training-testing values are plotted. For the PCA, a seven component approximation is made. To make a more quantitative comparison between these two approaches, and because both methods are reflectively invariant, the normalized least squares errors for each of these graphs is given in Table 1. The normalization used is *N*||**p**_*j*_||_1_/*n*, where *n* is the number of values in the training set and *N* the number in the testing set. It is seen, at least in this example, that the values using Eq (20) produce a uniformly better result than those obtained using a PCA. To make the point that the fit using a PCA varies with the scaling, the errors using two other scalings are also given in Table 1. The scaling used for PCA_1_ is considerably worse than the PCA values, while the PCA_2_ values in the last column are distinctly better.

**Table 1:** The E and PCA columns are the normalized least squares errors from Fig 7. The PCA_1_ and PCA_2_ columns are the errors for a PCA using two different column scalings.

	*E*	PCA	PCA_1_	PCA_2_
*p*_1_	3.92e−01	2.98e+00	6.14e+02	1.24e−01
*p*_2_	9.76e−02	2.97e+00	1.24e−01	8.65e−02
*p*_3_	2.13e−01	8.23e−01	1.13e+00	6.70e−01
*p*_4_	3.67e−01	1.41e+00	2.27e+00	1.94e+00
*p*_5_	1.88e−01	7.78e−01	2.36e+00	7.44e−02
*p*_6_	3.33e−02	2.23e−02	1.85e−01	2.19e−02
*p*_7_	1.07e−01	6.92e−02	3.55e+01	5.80e−02
*p*_8_	5.80e−02	3.58e−02	3.55e−01	3.64e−02

### Wine quality data

**Fig 8:**
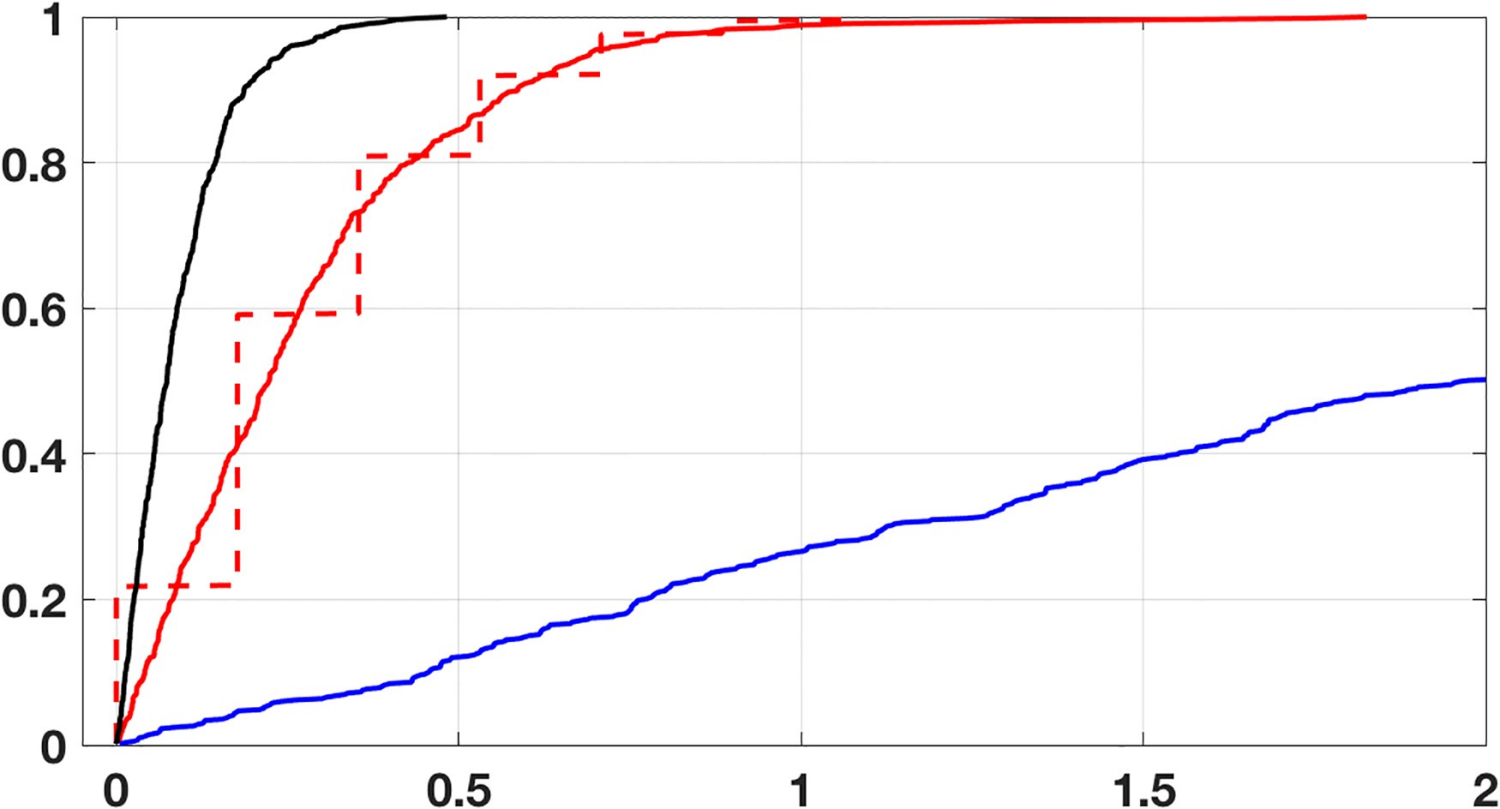
The regression error characteristic curve (REC) for the red wine testing data using the error function in Eq (20), solid red, using a PCA, blue, and least squares, black. The dashed red curve is the curve using Eq (20) after converting back to the original integer scale reported in [17].

As a second example, data mining has been used to predict tasting preferences for wine [17]. The dataset for red wine consists of 1599 instances (vectors) containing values for 12 attributes, 11 being physicochemical properties of the wine and one the quality score for taste. The physicochemical properties in this case are: fixed acidity (*α*_1_), volatile acidity (*α*_2_), citric acid (*α*_3_), residual sugar (*α*_4_), chlorides (*α*_5_), free sulfur dioxide (*α*_6_), total sulfur dioxide (*α*_7_), density (*α*_8_), pH (*α*_9_), sulphates (*α*_10_), and alcohol (*α*_11_). Following the protocol used in [17], the training set consists of 2/3 of the original (randomly chosen), and the testing set the remaining 1/3. They also used a regression error characteristic (REC) curve, which is defined in [18], to evaluate how well various regression procedures predict the taste score. To compare how Eq (20) does, using the training set, the minimizer is computed with the modified Polak-Ribière method described earlier, treating the data as defining a smooth function. The resulting REC curve determined using Eq (20) is shown in Fig 8. Also shown are the curves obtained using a PCA as well as from using a standard multivariable least squares regression. The dashed curve is what is obtained using Eq (20) if the predicted values are converted back into integer scores as originally used in [17]. Finally, for the two and three dimensional examples considered earlier, it was found that the centroid of the error simplex furnished a fairly accurate approximation for the minimizer. A measure of how close they are is given in Eq (18). Generalizing this formula for the wine example, it is found that *C*_*F*_ ≈ 0.15, which indicates they are reasonable close.

It is evident from Fig 8 that standard least squares provides better predictive values for the taste score than when using Eq (20). This can be quantified using the mean absolute deviation (MAD), which is defined as ||**y** − y*||_1_/*N*, where y* are the test values, **y** are the predicted values, and *N* is the number of observations in the testing set (note that the values are unscaled). For Eq (20) the MAD value is 1.5, while using least squares it is 0.5. Consequently, if given a particular bottle of red wine, least squares would provide a better predictor of how it tastes. What Eq (20) provides is a better model for how to modify the physicochemical properties to achieve a particular taste score, and the reason is reflective invariance. To illustrate, with the coefficients computed previously, the resulting MAD values for the other possible training-testing cases are given in Table 2. The fact that the least squares values are so poor is not surprising, and the reason was given earlier. Namely, the values obtained using *p*_*m*_ as the dependent variable are not equivalent to the values obtained if the model equation is solved for, say, *p*_1_, and then considering it as the dependent variable.

**Table 2:** The MAD values for red wine, as determined using Eq (20) and from conventional least squares. The last column is the ratio of the least squares value to the one obtained using Eq (20).

attribute	*E*	Least Squares	Ratio
fixed acidity	6.400e-01	7.455e+00	11.6
volatile acidity	2.299e-01	5.325e-01	2.3
citric acid	1.783e-01	2.481e+00	13.9
residual sugar	1.544e+00	8.459e+00	5.5
chlorides	8.297e-02	3.099e-01	3.7
free sulfur dioxide	1.241e+01	1.062e+02	8.6
total sulfur dioxide	3.848e+01	1.470e+02	3.8
density	9.008e-04	8.729e-03	9.7
pH	1.068e-01	3.286e+00	30.8
sulphates	3.350e-01	5.486e-01	1.6
alcohol	1.123e+00	2.075e+00	1.8
taste	1.493e+00	5.134e-01	0.3

### Wave height data

**Fig 9:**
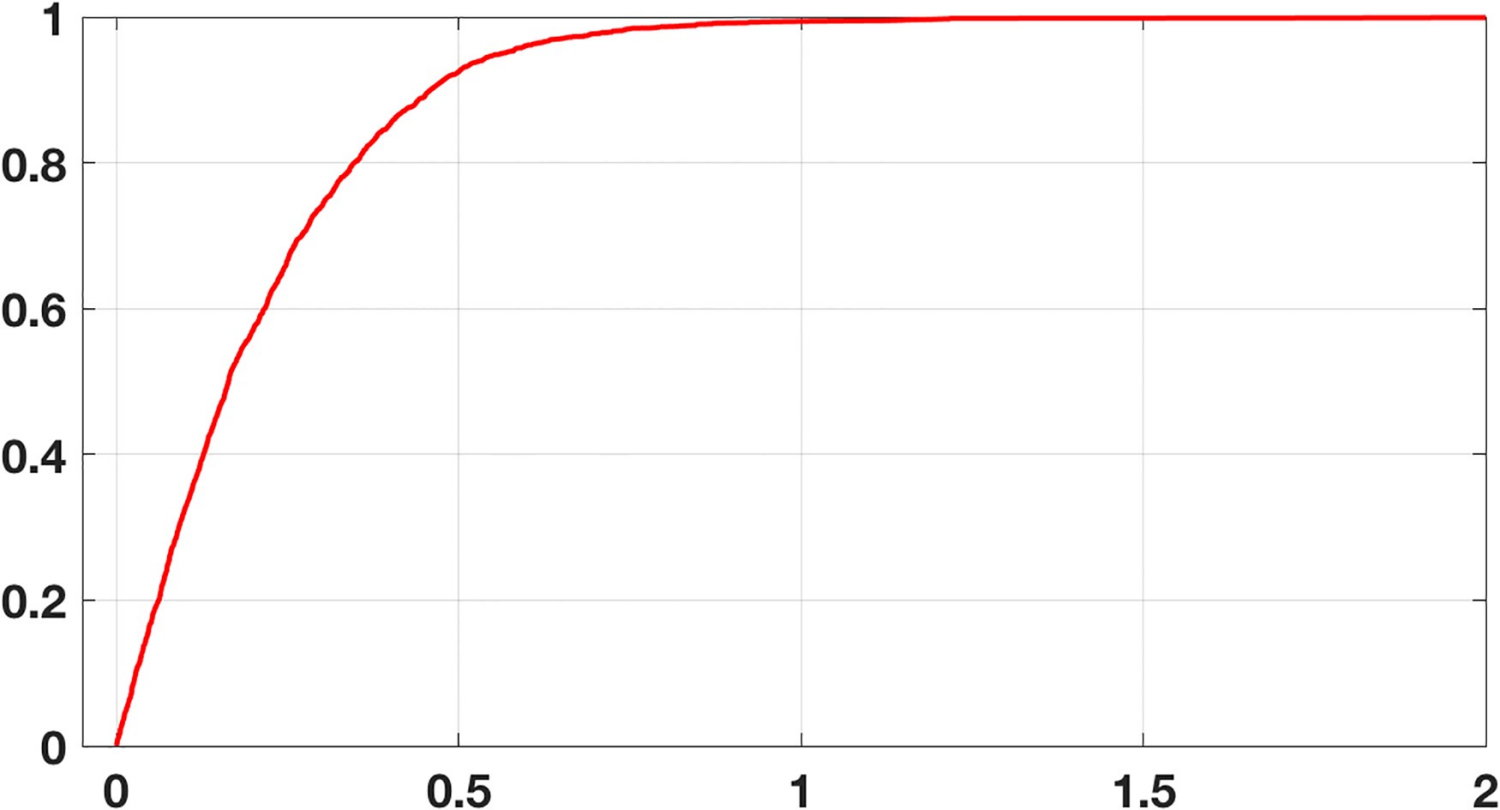
The regression error characteristic curve for the wave height data using the error function in Eq (20).

Wave height at Buoy Station 46006, located in the northern Pacific Ocean, is measured hourly, along with the wind direction, wind speed, wind gust, dominant wave period, average wave period, barometric pressure, and water temperature [19]. The specific data considered here come from measurements over approximately 11 months. The dataset consists of 7960 instances containing values for the eight attributes. A reduced version of this dataset was examined in [20], in comparing how various regression procedures do on real ecological data. The reduction was to use the daily average values rather than the hourly data, but all of the values are considered here. Following the protocol used in [20], the training set consists of 3/4 of the original (randomly chosen), and the testing set the remaining 1/4. The comparison was made using the mean squared prediction error (MSPE), which is defined as $||y-y^{*}{\left|\right|}_{2}^{2}/n$, where y* are the test values for the wave height, **y** are the predicted values, and *n* is the number of observations in the testing set (note that the values are unscaled). Using Eq (20), the MSPE is about 1.2, which compares favorability with the mean MSPE of 12.3 found in [20]. The resulting MAD value is about 0.8, and the REC curve is shown in Fig 9. Finally, for this example, *C*_*F*_ ≈ 0.16. This indicates that the centroid of the error simplex and the minimizer are close, although not as close as in the earlier examples.

### Chemical reaction data

The chemical oscillator known as the Belousov-Zhabotinskii reaction can be described with the following five reactions [21]

$$\begin{array}{cc} A+Y & \to X+P,\\ X+Y & \to 2P,\\ A+X & \to 2X+2Z,\\ 2X & \to A+P,\\ Z & \to \frac{1}{2}fY. \end{array}$$

The chemicals here are bromous acid (X), bromide (Y), cerium-4 (Z), bromate (A), and a product P. Given known initial concentrations for each species, measurements are made at later times to follow the evolution of the overall reaction. The complication is that one or more of these reactions are very fast, or occur at very low concentrations, so accurate measurements are difficult. What is demonstrated here is how the hyperplane fit can be used in the case of when four of the five concentrations are measured, and the fifth is determined using regression. What is important is that no matter which four are chosen, that the same (equivalent) value is obtained for the fifth species. To demonstrate, the reactions were run for 20 different initial concentrations, and the values for the five species were recorded at 60 second intervals up to 10 minutes. The resulting dataset consists of 180 instances, and these were randomly split into a training set (3/4) and a testing set (1/4). The values fitted are those for *Z*. Using Eq (20), the MAD value is 0.015 and the MSPE is 4.6 × 10^−4^. Using a standard least squares fit the values are 0.012 and 3 × 10^−4^, respectively. However, if you fit the values for, say, *A*, and then transform back to the equation for *Z*, then the MAD and MSPE values using Eq (20) remain unchanged while the least squares values are 0.024 and 10^−3^. As a final comment, using a reflectively invariant error function with chemical density fits has the distinct advantage of being able to determine possible conservation laws inherent in the system.

### Chlorophyll-*a* data

**Fig 10:**
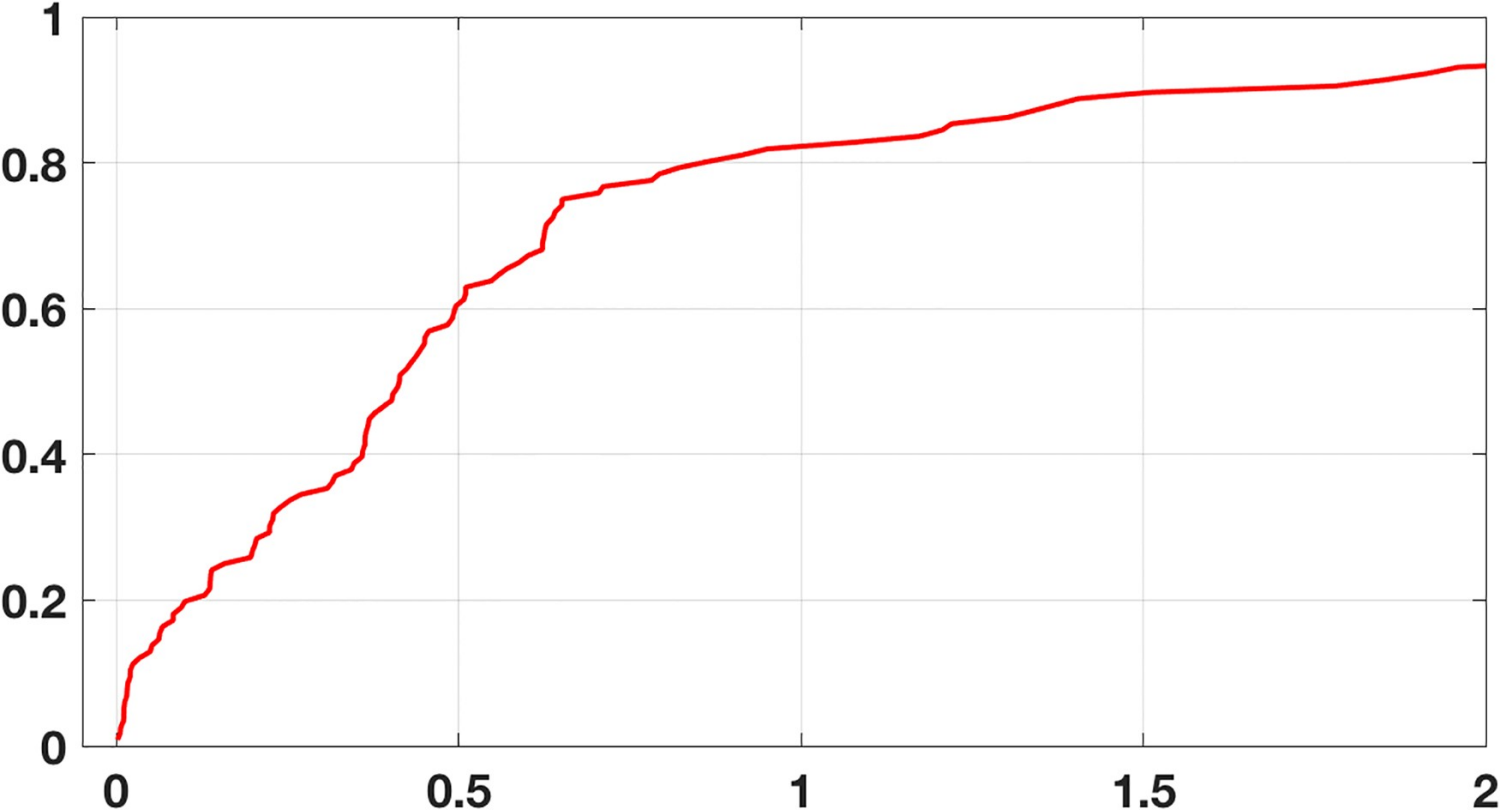
The regression error characteristic curve for the Chlorophyll-*a* data using the error function in Eq (20).

The link between phytoplankton and water chemistry has been the subject of several recent studies, although the connections with specific chemical species are incompletely understood. Several correlations have been made, and the physicochemical parameters most commonly considered are oxygen, pH, NH_4_-N (ammonium nitrogen), NO_3_-N (nitrate nitrogen), and PO_4_-P (phosphate phosphorus) [20, 22–24]. The latter study used the values for the Chlorophyll-*a* density, along with various chemical properties, of lakes in the Northeast that were measured over a four year period [25]. Altogether, there are 20 useable variables in this study, and 500 observations. After removing incomplete entries and others that are not useable, the data set consists of 348 observations. The model requires specification of which variables to use, and after some analysis of the data it comes down to five: total dissolved aluminum, nitrate, total nitrogen, total phosphorous, total suspended solids, and turbidity. In comparison, in [20], 10 variables were initially used. Using Eq (20), the resulting MSPE is about 1.1, which compares favorability with the mean MSPE of 2.4 found in [20]. The resulting normalized MAD value is about 0.7, and the REC curve is shown in Fig 10. Finally, for this example, *C*_*F*_ ≈ 0.13. This indicates that the centroid of the error simplex and the minimizer are close, similar to what was found for the wave height example.

## Other dimensional fits

It is possible to write down the formulas for the other cases, but it is more informative to consider a particular case that illustrates how this is done. So, consider the case of when there are four variables, *x*, *y*, *z*, and *w*. There are three subspace approximations possible, corresponding to one, two and three dimensions. The one and three dimensional cases were discussed above, and so only the two dimensional case is considered. Writing the model functions as *z* = *α*_11_*x* + *α*_12_*y*, *w* = *α*_21_*x* + *α*_22_*y*. This can be written in matrix form as

$$\begin{array}{c} (\begin{array}{c} z\\ w \end{array})=A(\begin{array}{c} x\\ y \end{array}). \end{array}$$

It is possible to rewrite the above equation in five different, but equivalent, ways. For example, if *z* and *w* are taken to be independent then

$$\begin{array}{c} (\begin{array}{c} x\\ y \end{array})=B(\begin{array}{c} z\\ w \end{array}), \end{array}$$

while if *y* and *z* are taken to be independent then

$$\begin{array}{c} (\begin{array}{c} x\\ w \end{array})=C(\begin{array}{c} y\\ z \end{array}). \end{array}$$

The other three forms are

$$\begin{array}{c} (\begin{array}{c} y\\ w \end{array})=D(\begin{array}{c} x\\ z \end{array}),\;(\begin{array}{c} y\\ z \end{array})=E(\begin{array}{c} x\\ w \end{array}),\;\text{and}\;\;(\begin{array}{c} x\\ z \end{array})=F(\begin{array}{c} y\\ w \end{array}). \end{array}$$

The entrees in the above matrices are known expressions involving the original coefficients *α*_11_, *α*_12_, *α*_21_, and *α*_22_. For example,

$$\begin{array}{c} B=A^{-1}=\frac{1}{\alpha_{11}\alpha_{22}-\alpha_{12}\alpha_{21}}(\;\begin{array}{cc} \alpha_{22}\;\; & \;-\alpha_{21}\\ -\alpha_{12}\;\; & \;\alpha_{11} \end{array}\;). \end{array}$$

The error function corresponding to the variable *x* is

$$\begin{array}{c} E_{x}=\sum_{i=1}^{n}[{\left(x_{i}-B_{11}z_{i}-B_{12}w_{i}\right)}^{2}+{\left(x_{i}-C_{11}y_{i}-C_{12}z_{i}\right)}^{2}+{\left(x_{i}-F_{11}y_{i}-F_{12}w_{i}\right)}^{2}]. \end{array}$$

The three terms in the above sum correspond to the case when *x* is taken to be dependent. In a similar manner,

$$\begin{array}{c} E_{y}=\sum_{i=1}^{n}[{\left(y_{i}-B_{21}z_{i}-B_{22}w_{i}\right)}^{2}+{\left(y_{i}-D_{11}x_{i}-D_{12}z_{i}\right)}^{2}+{\left(y_{i}-E_{11}x_{i}-E_{12}w_{i}\right)}^{2}]. \end{array}$$

The corresponding error to determine the *α*’s is

$$\begin{array}{c} E\left(\alpha_{11},\alpha_{12},\alpha_{21},\alpha_{22}\right)={\left(E_{x}E_{y}E_{z}E_{w}\right)}^{1/4}. \end{array}$$

## Concluding remarks

The principal conclusions from this study are:

1. Scale and rotational invariance of the error function are incompatible.
2. Using the geometric mean of the ordinary least squares error functions, one obtains an error function which is scale and reflectively invariant, and which is easily extendable to low dimensional approximations for multilinear regression. For the two cases worked out, which correspond to a line and hyperplane approximation, the minimizer can be well approximated using the centroid of the error simplex obtained from the minimizers for the ordinary least squares error functions.

Because the error function used here is not quadratic, finding the minimizer requires using a nonlinear optimization procedure. The result is that more computational time is needed than for linear least squares. How much time depends on the size of the data matrix, and the number of variables involved. For the wine example considered earlier (12 variables and 1599 data vectors), linear least squares using MATLAB’s *mldivide* routine takes about 1 msec, while the nonlinear optimization procedure takes about 100 msec (using a 2017 iMac). So, although the relative time is fairly large, the actual time is small. The proposed error function does have the advantage of having a warm start, which is the centroid approximation, and this helps reduce the computing time.

In terms of future work, it remains to determine if the centroid approximation applies to the other lower dimensional approximations. Also, there is the question as to the sensitivity of the minimizer to outliers in the training set. This is a problem for a PCA, and one approach to improve the robustness of a PCA is to switch from a *ℓ*_2_-norm to a *ℓ*_1_-norm [26, 27], a *ℓ*_*p*_-norm [28], or a norm based on a generalized mean [29]. These are used, in part, because they preserve the rotational invariance of a PCA. It is straightforward to use these norms with the geometric mean function, and this will not affect its invariance properties. What has not been investigated is the sensitivity of the proposed error function to outliers, or whether switching norms might reduce any potential sensitivities.
